# Long COVID: plasma levels of neurofilament light chain in mild COVID-19 patients with neurocognitive symptoms

**DOI:** 10.1038/s41380-024-02554-0

**Published:** 2024-04-27

**Authors:** Elisa Gouvea Gutman, Andreza Lemos Salvio, Renan Amphilophio Fernandes, Larissa Araujo Duarte, Jessica Vasques Raposo-Vedovi, Helena França Alcaraz, Milene Ataíde Teixeira, Giselle Fazzioni Passos, Karoline Queiroz Muniz de Medeiros, Mariana Beiral Hammerle, Karina Lebeis Pires, Claudia Cristina Ferreira Vasconcelos, Luciane Almeida Amado Leon, Cláudia Pinto Figueiredo, Soniza Vieira Alves-Leon

**Affiliations:** 1 Translational Neuroscience Laboratory (LabNet), Biomedical Institute, Federal University of the State of Rio de Janeiro/UNIRIO, Rio de Janeiro, RJ ZIP CODE 20211-040 Brazil; 2 Clinical Medicine post-graduation program, Federal University of Rio de Janeiro, Rio de Janeiro, RJ Brazil; 3 School of Pharmacy, Federal University of Rio de Janeiro, Rio de Janeiro, RJ Brazil; 4 Department of Neurology, Clementino Fraga Filho University Hospital, Federal University of Rio de Janeiro, Rio de Janeiro, RJ Brazil; 5 Division of Neurology, Gaffrée and Guinle University Hospital, Federal University of the State of Rio de Janeiro/UNIRIO, Rio de Janeiro, RJ Brazil; 6 Laboratório de Desenvolvimento Tecnológico em Virologia. IOC/FIOCRUZ, Rio de Janeiro, Brasil

**Keywords:** Prognostic markers, Neuroscience, Biological techniques

## Abstract

It is well known the potential of severe acute respiratory coronavirus type 2 (SARS-CoV-2) infection to induce post-acute sequelae, a condition called Long COVID. This syndrome includes several symptoms, but the central nervous system (CNS) main one is neurocognitive dysfunction. Recently it has been demonstrated the relevance of plasma levels of neurofilament light chain (pNfL), as a biomarker of early involvement of the CNS in COVID-19. The aim of this study was to investigate the relationship between pNfL in patients with post-acute neurocognitive symptoms and the potential of NfL as a prognostic biomarker in these cases. A group of 63 long COVID patients ranging from 18 to 59 years-old were evaluated, submitted to a neurocognitive battery assessment, and subdivided in different groups, according to results. Plasma samples were collected during the long COVID assessment and used for measurement of pNfL with the Single molecule array (SIMOA) assays. Levels of pNfL were significantly higher in long COVID patients with neurocognitive symptoms when compared to HC (*p* = 0.0031). Long COVID patients with cognitive impairment and fatigue symptoms presented higher pNfL levels when compared to long COVID patients without these symptoms, individually and combined (*p* = 0.0263, *p* = 0.0480, and 0.0142, respectively). Correlation analysis showed that levels of cognitive lost and exacerbation of fatigue in the neurocognitive evaluation had a significative correlation with higher pNfL levels (*p* = 0.0219 and 0.0255, respectively). Previous reports suggested that pNfL levels are related with higher risk of severity and predict lethality of COVID-19. Our findings demonstrate that SARS-CoV-2 infection seems to have a long-term impact on the brain, even in patients who presented mild acute disease. NfL measurements might be useful to identify CNS involvement in long COVID associated with neurocognitive symptoms and to identify who will need continuous monitoring and treatment support.

## Introduction

The SARS-CoV-2 pandemic had worldwide devastating effects, and the late consequences of the disease are still emerging as a public health concern. Besides several acute complications, COVID-19 patients may also experience persistent sequelae, collectively termed long COVID [1]. According to WHO [2], long COVID can be defined as a condition that occurs in individuals with a history of probable or confirmed SARS-CoV-2 infection, with symptoms that last for at least 2 months, usually 3 months from the onset of COVID-19 and cannot be explained by an alternative diagnosis.

This syndrome includes various neurocognitive and other neurological signs and symptoms that could have a major impact on return to everyday activities and quality of life [3]. The symptoms may fluctuate or relapse over the time and have been registered immediately or soon after the recovery period from an acute COVID-19 episode or it can persist since the initial illness [2].

The occurrence of neurological complications is expected in hospitalized COVID-19 patients, and it includes high frequency of encephalopathy and other neurological manifestations in the acute disease phase, due to the multiorgan damage caused by SARS-CoV-2 [4–6]. However, those with mild initial COVID-19 disease who never required hospitalization also often develop long COVID [7].

Long COVID evolves a series of not yet well-defined neurological symptoms. The most frequents has been a spectrum of cognitive symptoms, chronic fatigue, and neuropsychiatric complaints such as new-onset depression and anxiety. It also includes headaches, dizziness, disorders of smell and taste, among others [8, 9]. The prevalence of long COVID symptoms vary from 30-86% of post-COVID-19 individuals within 6 months of the acute phase of the SARS-CoV-2 infection, depending on disease severity [10–12].

Several mechanisms have been proposed to explain the neuropathogenesis of long COVID, including active viral infection on the central nervous system (CNS) immune activation secondary to systemic inflammatory responses, spike protein’s damage to the endothelium and perivascular inflammation microvascular injuries, or hypoxic consequences of severe disease [13, 14]. Nevertheless, there are many questions and dubiousness that remains unknown, related to long COVID, such as duration of symptoms, disease mechanism, phenotypes that are grouped under the term long COVID, and the most troubling for the patients, the risk of serious and prolonged sequelae [15].

Plasma neurofilament light chain (pNfL) is a highly specific structural protein of neurons and it has been validated as a biomarker for neuroaxonal damage [16, 17]. The tight correlation between levels in cerebrospinal fluid (CSF) and blood samples, serum and plasma [18], make it widely usable as a biomarker for neuroinflammation and degeneration in a series of neurological conditions, such as Multiple Sclerosis and Alzheimer’s Disease [16, 19, 20], and as a predictor for neurological outcome in the intense care unit (ICU) [21, 22].

Several studies were able to associate increase in levels of pNfL with CNS injury in the acute phase of both severe and mild-to-moderate COVID-19, indicating the possible neuroaxonal injury that occurs in acute SARS-CoV-2 infection [23, 24]. Changes in these CNS damage biomarkers have been associated with neuropsychiatric complications, such as encephalopathy and delirium, severity of COVID-19, poor disease outcome, and even death [25–32]. In this sense, it has been useful for clinicians worldwide, enabling a more guided approach, in the way precision medicine works.

In this study, we investigate whether there is evidence of CNS injury, here indicated by pNfL, in long COVID patients with chronic neurocognitive symptoms after mild disease, thereby measuring the importance of pNfL as a biomarker of brain injury in non-hospitalized long COVID patients.

## Methods

### Participants and study design

The study was conducted with a group of long COVID patients from Clementino Fraga Filho University Hospital and Gaffrée and Guinle University Hospital, in Rio de Janeiro, Brazil, between December 11, 2020, and December 20, 2022. It encompassed patients with mild acute COVID-19.

We used the same cohort from our previous study paper entitled “SARS-CoV-2 Spike protein induces TLR4-mediated long-term cognitive dysfunction recapitulating post-COVID-19 syndrome in mice”. In that study, our group showed that Spike-induced cognitive impairment in mice triggers innate immunity activation through TLR4, culminating in microgliosis, neuroinflammation, and synaptic pruning [33]. Remarkably, we also found plasma NfL increase in mice with Spike-induced cognitive impairment and this mechanism was dependent on TLR4 activation, because early TLR4 inhibition mitigated changes in NfL levels.

We validated our preclinical findings by examining whether TLR4 genetic variants could be associated with poor cognitive outcome in patients with COVID-19 with mild disease. In a cohort of patients with mild COVID-19 carrying the GG genotype of the TLR4-2604G > A (rs10759931) variant, we identified increased expression of TLR4 and high risk for cognitive impairment after SARS-CoV-2 infection compared with the GA genotype. In the current study, the same patients were used to validate the NfL results, previously reported by us in mice, in humans. So, we now investigate the neurological and psychiatric consequences of long COVID in a more direct approach, comparing the NfL levels in these same patients with the results of the neuropsychological exams.

For the pNfL analysis, it included 32 matched health control (HC) subjects who have never been infected with SARS-CoV-2, from a blood bank of HC with samples collected before the pandemic.

Inclusion criteria involved: COVID-19 diagnosis was confirmed with polymerase chain reaction; accordance with the WHO definition of post-COVID-19 syndrome (have the assessment at least three months after the end of acute symptoms, and duration of symptoms of at least two months) [2]; patient’s acute clinical status of mild illness, according to WHO definition of the clinical spectrum of SARS-CoV-2 infection [32]; agreement to collect blood samples for analysis and to perform the neuropsychiatric evaluation.

Exclusion criteria encompassed: individuals with 18-years-old or younger, individuals with 60 years-old or more, moderate or severe acute COVID-19 course according to WHO definition of the clinical spectrum of SARS-CoV-2 infection [32], and/or previously known cognitive impairment or other neuropsychiatrist disorders that could interfere with the test results.

All of them were evaluated from a clinical point of view, with detailed clinical history, social and epidemiological information. The post-COVID-19 assessment included comorbidities, symptoms experienced during acute infection, date of symptom onset, and long COVID persistent symptoms, including fatigue, persistence of anosmia and dysgeusia, and neurocognitive symptoms.

### Neurocognitive assessment

All participants infected by SARS-CoV-2 underwent neuropsychological testing, which included: symbol digit modalities test (SDMT), fatigue severity scale (FSS) and hospital anxiety and depression scale (HADS).

The SDMT [34] is a validated test to identify cognitive impairment through a straightforward assignment that comprises attention, processing speed and motor skills. The test page presents a series of nine different symbols, each one paired with a single digit labeled 1–9 and a list of the same symbols below, with blank spaces instead of numbers. The patient must manually fill the blank space under each symbol with the corresponding number over a 90 s period, with the number of total items, correct pairings, and errors registered by the evaluator [35, 36].

Demographically influenced T-scores for each participant were calculated based on multiple regression equations derived from the healthy group’s scores, described by Parmenter et al. 2010 [37]. The raw score of the SDMT is converted to scaled scores (M = 10, SD = 3) using the cumulative frequency distribution of the test. This served to normalize all the test score distributions. Then, the resulting scaled scores are regressed on age, age-squared, sex, and education.

Next, the participants’ raw test scores are converted to scaled scores using the raw-to-scale-score conversions, derived from the healthy controls. Multiple regression equations derived from the healthy controls are applied to compute demographically predicted scores for each participant. These predicted scores were then subtracted from each participant’s actual scores and the differences were divided by the standard deviation of the controls group’s raw residuals for each measure. Finally, the resulting values were converted to T scores. To increase clinical interpretation, the calculated cut-points are one standard deviation below the mean (*T* ≤ 40) to indicate impairment.

The FSS is a 9-item self-reported questionnaire developed to assess fatigue in patients with systemic lupus erythematosus and multiple sclerosis [38], by asking subjects to choose for each item the number from 1 to 7 which best applied to them [38, 39]. 1 indicates strongly disagree and 7 indicates strongly agree. Its use has expanded to several diseases that present with chronic fatigue, including long COVID [40–43]. We employed the standard cut-off (FSS mean score ≥ 28) to determine clinically significant levels of fatigue [39].

The HADS is a self-reported scale developed to identify the presence of anxiety disorders and depressive symptoms in people in nonpsychiatric hospital clinics [44], however it has good psychometric properties of both the anxiety and depression scales for assessing anxiety and depressive symptoms within the general population and in other disorders, including COVID-19 patients [45–48]. It is composed of 14 items, subdivided into HADS-A and HADS-D. HADS-A consists of 7 items assessing anxiety symptoms whereas HADS-D consists of 7 items evaluating depressive symptoms. Each item is scored on a 4-point scale (0–3) providing a maximum of 21 points for each subscale [46]. It employed a cut-off score of ≥8 points for each scale since this value has shown good sensitivity and specificity to determine the presence of anxiety or depressive symptoms [48, 49].

Based on the neurocognitive tests results, study subjects were subdivided into the following groups, according to each test classification:

1. SDMT: Long COVID patients with cognitive impairment and long COVID without cognitive impairment.
2. FSS: Long COVID patients with fatigue and long COVID patients without fatigue.
3. HADS-A: Long COVID patients with anxiety and long COVID patients without anxiety.
4. HADS-D: Long COVID patients with depression and long COVID patients without depression.

### Ethics

This work was approved by the Brazilian Ethics Committee (CONEP, CAAE 33659620.1.1001.5258). All subjects signed the informed consent term, agreeing to participate in this research.

### Procedures

Blood samples were collected in EDTA tubes at the moment of the post-COVID-19 assessment, the same day as the neuropsychologic test. The samples were processed by centrifugation at 2880*g* and 4 °C for 15 min to separate the buffy coat from plasma. Both plasma and buffy coat were frozen in aliquots at −80 °C. For NfL analysis, it was used the plasma from the samples.

Plasma NfL measurement was performed at the Translational Neuroscience Laboratory of the Federal University of the State of Rio de Janeiro, Brazil, using commercially available single molecule array (SIMOA) assays on an SR-X Analyzer, with the plasma NfL kit, as described by the manufacturer (Quanterix, Billerica, MA). Calibrators were run in duplicates, while samples were diluted four-fold and run in singlicates. Two quality control (QC) samples with different levels were run in duplicates at the beginning and the end of each run. Repeatability and intermediate precision were both 8.7% for the QC sample with an pNfL concentration of 8.4 pg/mL and 5.9% for the 79.6 pg/mL sample.

First, we compared the levels of pNfL of all patients that had at least one of the four tests altered with the HC group control, to assess the relationship between CNS damage and long COVID symptoms. It is well established that pNfL concentration is correlated with aging, and therefore, it was determined that the HC had a similar age distribution from the patients’ groups. The mean (IQR) and median age of HC were, respectively, 34.79 (25–41) and 35. All patients and controls with less than 18 years and more than 59 were excluded.

Then, we compared the levels of pNfL of the subgroups of each test to understand their influence on the possible CNS damage individually. At last, we investigated the correlation between each test result and the pNfL levels.

### Statistical analyses

Statistical analysis data were summarized as number of patients (percentage/ frequency), normally distributed variables as mean (SD), or median (interquartile range) for non-normally distributed variables. Each of these groups was compared with control group using one-way ANOVA followed by the Kruskal–Wallis test by for data without Gaussian distribution. Followed by Dunn’s multiple comparison test to find out which means are significantly different. The non-parametric Mann–Whitney *U* test was applied to determine whether two groups of symptomatic long COVID patients and without symptoms and or healthy controls were statistically different for non-parametric variables. Associations between quantitative variables were assessed using the Pearson correlation test. A *p*-value < 0·05 was considered statistically significant. Graphs and corresponding statistical analyses were generated using Prism (GraphPad Software version 9.5.1, La Jolla, CA, USA).

## Results

### Post-COVID-19 patients: demographics and study groups

A total of 63 post-COVID-19 volunteers with mild acute COVID-19 were recruited for inclusion in this study. The sociodemographic characteristics are summarized in Table 1. The mean (IQR) age in the COVID-19 cohort was 39.21 (19–57); 49 (77.8%) were female. Most of the patients (37; 58.7%) did not have any known comorbidity. Among the comorbidities, the most prevalent was hypertension (14; 22.2%), followed by obesity (9;14.3%).

**Table 1:** Sociodemographic characteristics, acute and long COVID symptoms of the post-COVID-19 volunteers.

Sociodemographic			
Category		Mean	Range
Age		39.21	(19–57)
Category		Frequency	Percentage
Genre	Female	49	77.8%
	Male	14	22.2%
Color	White	38	60.3%
	Brown	13	20.6%
	Black	10	15.9%
	Yellow	2	3.2%
No. of comorbidities	0	37	58.7%
	1	16	25.4%
	2	7	11,1%
	3	3	4.8%
Type of comorbidity	Hypertension	14	22.2%
	Obesity	9	14.3%
	Diabetes	4	6.3%
	Asthma	2	3.2%
	Others	12	19.0%
Acute COVID-19 symptoms			
*Category*			
Fatigue		49	77.8%
Headache		38	60.3%
Anosmia		33	52.4%
Ageusia		33	52.4%
Cough		33	52.4%
Fever		30	47.6%
Coryza		26	41.3%
Inappetence		24	38.1%
Myalgia		23	36.5%
Sore throat		22	34.9%
Diarrhea		19	30.2%
Arthralgia		18	28.6%
Dizziness		14	22.2%
Emesis		8	12.7%
Subjective post-COVID-19 symptoms			
*Category*		Mean	Range
Interval between the onset of COVID-19 and the long-COVID assessment (in months)		8.01	(3–30)
*Category*			
Any		56	88.9%
Cephalgia		18	28.6%
Anosmia		16	25.4%
Dysgeusia		11	17.5%
Cacosmia		7	11.1%
Paresthesia		7	11.1%
Parosmia		7	11.1%
Dizziness		7	11.1%
Mialgia		4	6.3%

pNfL subgroups division according to neuropsychiatric assessment				
HC group		*N*	Mean age (IQR)	Median age
		35	34.79 (25–41)	35
*SDMT subgroups*	Frequency	Percentage		
Post COVID-19 patients with cognitive impairment	41	65.1%		
Post COVID-19 patients without cognitive impairment	22	34.9%		
*FSS subgroups*	Frequency	Percentage		
Post COVID-19 patients with fatigue	44	69.8%		
Post COVID-19 patients without fatigue	19	30.2%		
*HADS-A subgroups*	Frequency	Percentage		
Post COVID-19 patients with anxiety	33	52.4%		
Post COVID-19 patients without anxiety	30	47.6%		
*HADS-D subgroups*	Frequency	Percentage		
Post COVID-19 patients with depression	25	39.7%		
Post COVID-19 patients without depression	38	60.3%		

### Symptoms associated with acute COVID-19

The mean interval between the onset of COVID-19 and the long COVID assessment was 8.01 (3–30) months. All patients had some symptoms in the acute phase of the disease, however none of them were hospitalized, since it’s a sample composed of mild COVID-19 patients. The most frequent symptoms during the acute phase were fatigue (49; 77.8%), headache (38; 60.3%), anosmia (33; 52.4%) and ageusia (33; 52.4%). Acute COVID-19 symptoms frequency is also described in Table 1.

### Long COVID symptoms and neurocognitive assessment

The most frequently reported post-COVID-19 symptoms were fatigue (44; 69.8%), cognitive impairment (41; 65.1%), anxiety (33; 52.4%) and depression (25; 39.7%). As to objectify these symptoms, it was only considered patients with subjective complaints that started after COVID-19 and corresponding alterations on the neurocognitive assessment. Cognitive performance, fatigue, anxiety and depression were analyzed using, respectively SDMT, FSS, HADS-A and HADS-D. Others long COVID symptoms are described in Table 1.

### NfL levels according to neurocognitive assessment

**Fig. 1:**
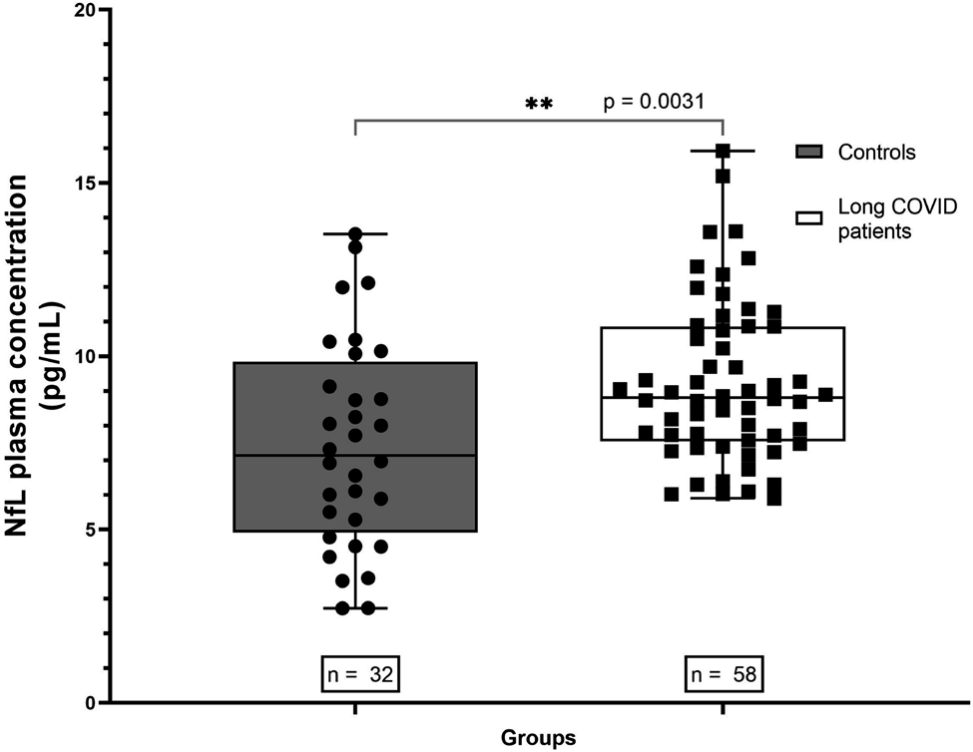
Comparison of pNfL levels between HC samples collected before the pandemic and long COVID patients with alteration of at least one neurocognitive test. The long COVID patients had significantly higher levels of pNfL (*p* = 0.0031).

**Fig. 2:**
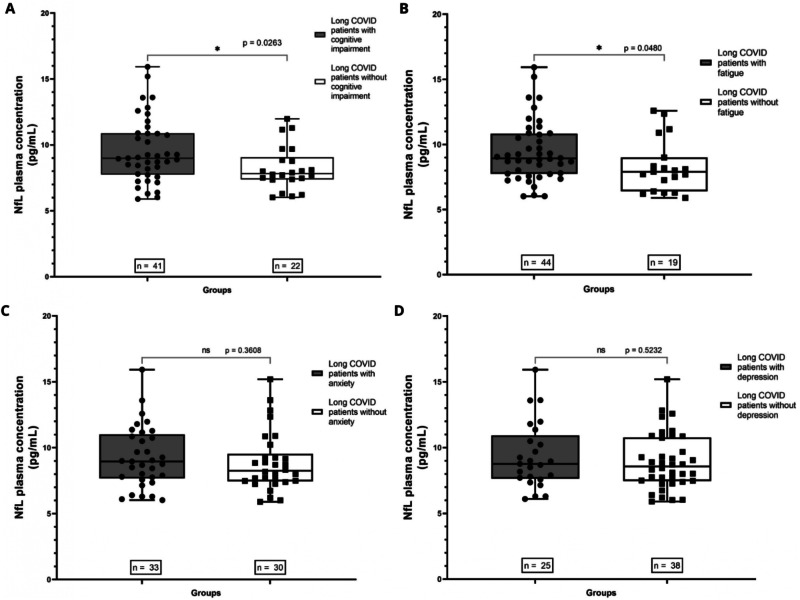
Comparison of pNfL levels (pg/mL) between neurocognitive assessment subgroups. **A** Long COVID patients with cognitive impairment and long COVID patients without cognitive impairment. **B** Long COVID patients with fatigue and long COVID patients without fatigue. **C** Long COVID patients with anxiety and long COVID patients without anxiety. **D** Long COVID patients with depression and long COVID patients without depression.

Long COVID patients with at least one of the four tests altered had significantly higher levels of pNfL when compared to the HC group (*p* = 0.0031) (Fig. 1). On the individual symptom’s evaluation, long COVID patients with cognitive impairment in the SDMT had significantly higher levels of pNfL when compared to long COVID patients without cognitive impairment (*p* = 0.0256) (Fig. 2A). Long COVID patients with fatigue in the FSS had significantly higher levels of pNfL when compared to long COVID patients without fatigue (*p* = 0.0480) (Fig. 2B).

Long COVID patients with anxiety in the HADS-A didn’t have significantly higher levels of pNfL when compared to long COVID patients without fatigue (*p* = 0.3608) (Fig. 2C). Similarly, long COVID patients with depression in the HADS-D did not have significantly higher levels of pNfL when compared to long COVID patients without depression (*p* = 0.5232) (Fig. 2D).

**Fig. 3:**
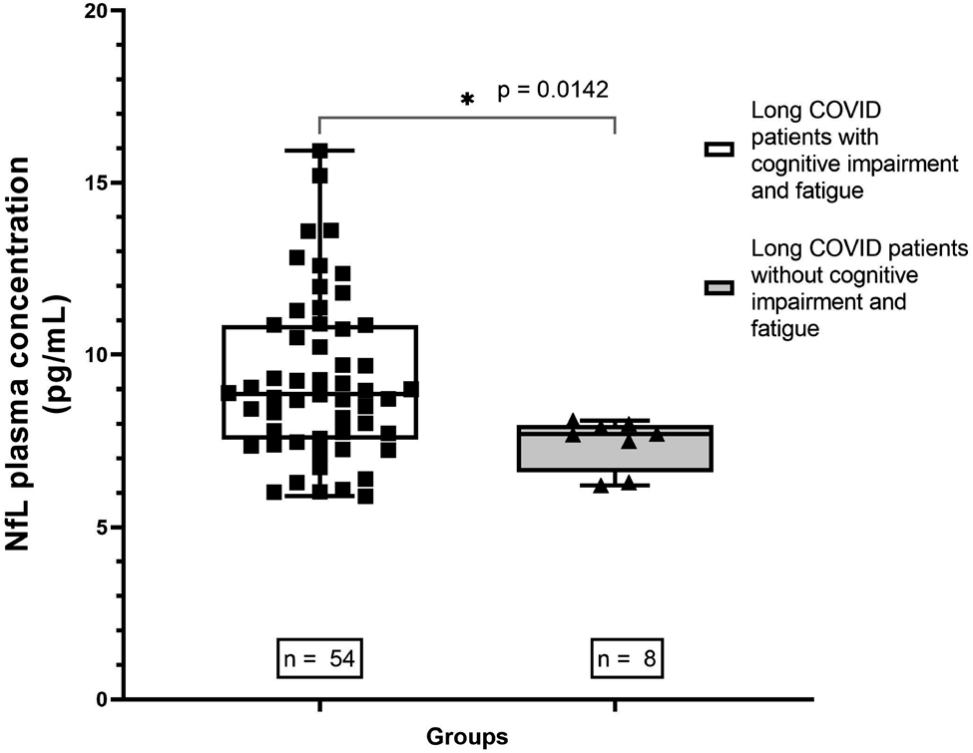
Comparison of pNfL levels (pg/mL) between long COVID patients with cognitive impairment and fatigue, and long COVID patients without cognitive impairment and fatigue. The first group had significantly higher levels of pNfL (*p* = 0.0031).

Since both cognitive and fatigue results were significant, it was made a second analysis, which included the comparison of pNfL levels between a group of long COVID patients with cognitive impairment and fatigue, and a group of long COVID patients without cognitive impairment and fatigue. The first group had significantly higher levels of pNfL (*p* = 0.0031) (Fig. 3).

**Fig. 4:**
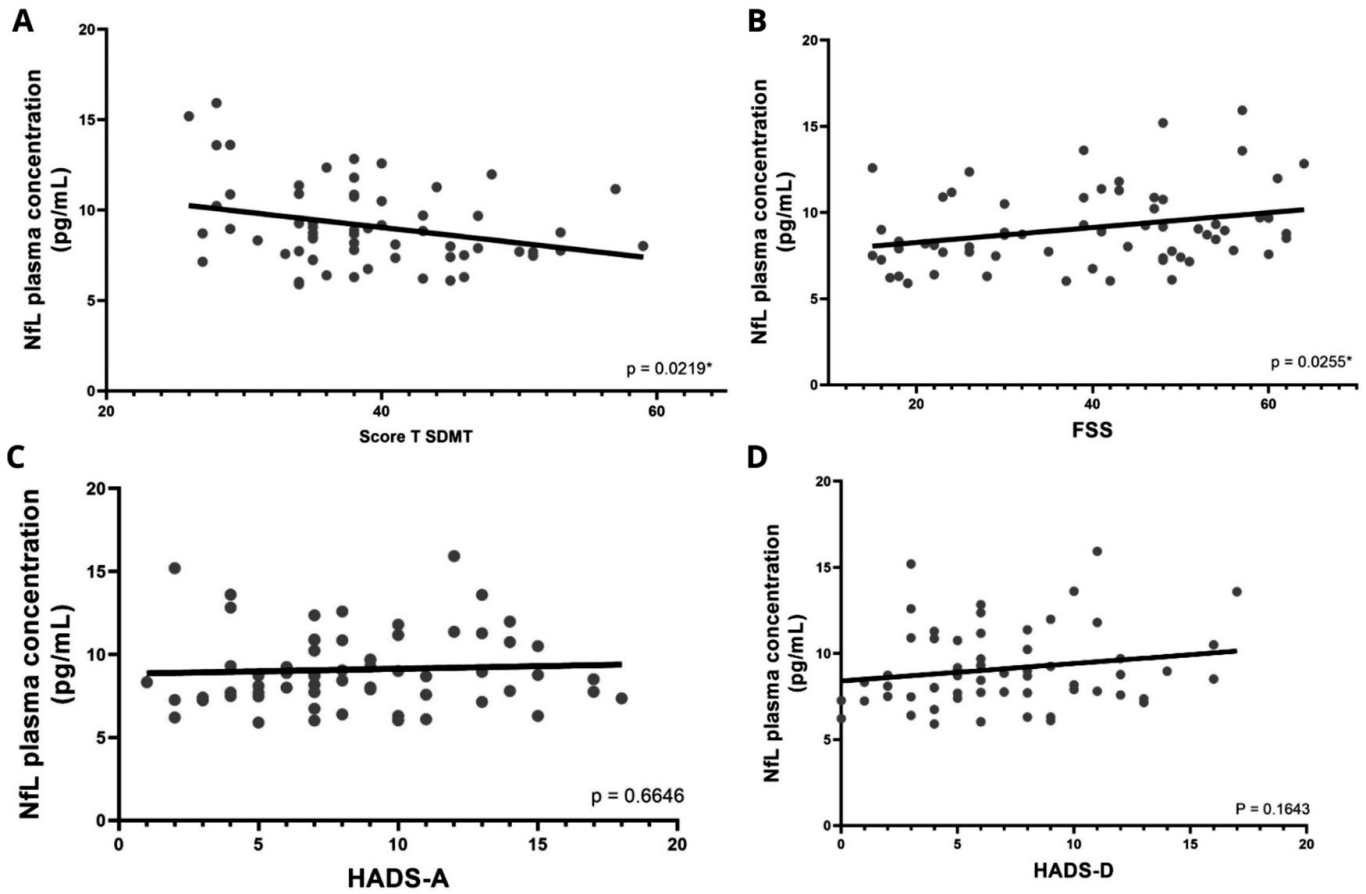
Correlation between pNfL levels (pg/mL) among neuropsychological exams. **A** Correlation between pNfL levels (pg/mL) and SDMT Score T results. **B** Correlation between pNfL levels (pg/mL) and FSS results. **C** Correlation between pNfL levels (pg/mL) and HADS-A results. **D** Correlation between pNfL levels (pg/mL) and HADS-D results.

On the correlation analysis, it was shown a significantly negative correlation between pNfL levels and the SDMT results (*p* = 0.0219) (Fig. 4A) and a significantly positive correlation between pNfL levels and the FSS results (*p* = 0.0255) (Fig. 4B). Thus, it can be inferred that lower SDMT results, namely, worse cognitive impairment, associate with greater CNS damage, manifested by higher pNfL levels. Likewise, higher FSS results, videlicet, worse fatigue, also associate with more CNS damage.

Conversely, pNfL levels did not correlate with HADS-A (*p* = 0.6646) or HADS-D (*p* = 0.1643) results (Fig. 4C, D, respectively). This goes accordingly with the test *t* results, that presented no difference between long COVID patients with anxiety and depression, and long COVID patients without anxiety and depression. These findings draw the inference that these symptoms, unlike cognitive impairment and fatigue, not interfere with ongoing CNS damage.

## Discussion

Despite the high frequency of neurologic involvement in hospitalized COVID-19 patients, there is a large amount of asymptomatic and mild symptomatic COVID-19 individuals who developed long COVID symptoms after the SARS-CoV-2 infection [1]. In this study, 77.8% of the sample was female. Despite being a relatively small sample, this finding is similar to previous studies that have identified the female gender with increased likelihood to develop long COVID syndrome when compared to their male counterparts [50, 51]. A multivariable analysis found that the female gender had a threefold higher risk to develop long COVID syndrome [52]. Interestingly, the same study presented those overall patients of female gender had a milder form of disease, while no association was found between severity of acute disease and long COVID.

These sex differences may in part be explained by immune response differences [53–56], as well as the disproportionate impact of the COVID-19 pandemic on the daily-life of the female genre compared to males [57]. Females seem to mount prompter and more efficient innate and adaptive immune responses in the early phase of disease, which may protect them and lead to a more favorable outcome on the acute state of COVID-19 [58]. Nonetheless, this same difference might as well portray a part in the perpetuation of long COVID manifestations [53, 54]. Furthermore, females appear to have worse long COVID outcomes associated with pandemic-related stressors and social determinants of health [57]. Female hormones may also play a role in perpetuating the hyperinflammatory status of the acute phase even after recovery [52, 59].

These neurological manifestations could reflect a nonspecific effect of respiratory viral infections on the CNS [60]. The underlying mechanism common to these different disease conditions might be represented by hypoxic damage due to respiratory insufficiency, thus resembling NfL increase following hypoxic-ischemic injury after cardiac arrest [22]. This theory is supported by the “happy hypoxia” seen in some COVID-19 patients, in which patient’s symptoms of dyspnea and signs of respiratory distress are absent [61]. Another plausible determinant might be the systemic hyperinflammatory state that promotes sepsis-associated encephalopathy [62].

The increased pNfL levels in long COVID could be compatible with both hypoxic and inflammatory injury, but there’s probably other mechanisms of COVID-19-induced neuronal impairment. Namely, a direct damage to the nervous tissue by SARS-CoV-2 [33, 63] or the entry of the Spike protein of the SARS-CoV-2 virus in the CNS, disrupting the blood-brain barrier (BBB), leading to neuro-inflammation and contributing to long COVID [14].

Additionally, some recent studies have shown similarities between SARS-CoV-2 protein sequences and human proteins found in multiple organs/tissues, including the nervous system, indicating the potential for cross-reactive immune recognition of these regions by T cells and antibodies produced by B cells, and the possible generation of multi-system autoimmune reactions [64, 65]. Notably, N-methyl-d-aspartate receptor (NMDAR), glutamic acid decarboxylase 65-kD isoform (GAD65), and myeloperoxidase may be involved not only in acute encephalitis or demyelinating events but also in neurocognitive and psychiatric manifestations, frequently seen in long COVID patients [66–68]. Pathological results in cognitive screening were associated with the presence of antibodies against NMDAR and GAD65 in CSF of long COVID patients [69]. Our group recently published an in silico analysis that showed molecular mimicry between these autoantibodies and SARS-CoV-2 antigens, which may be a prominent asset to understanding the pathogenesis of long COVID cognitive and psychiatric symptoms [70].

Another possible contributor to SARS-CoV-2 CNS damage is the cell senescence, characterized by a permanently arrested cell cycle that is no longer responsive to differentiation and apoptotic signaling processes [71]. Although mal-functioning, senescent cells continue to be metabolically active and are responsible for causing a hyperinflammatory state in the body, due to its senescence-associated secretory phenotype, accelerating age-related neurodegenerative processes [72].

SARS-CoV-2 seems to trigger *ferrosenescence*, a phenomenon known as “neurodegeneration-by-iron” - premature molecular aging due to iron-induced damage to both DNA and the genomic repair systems, especially the p53 [73, 74]. The virus upregulates intracellular iron and Ca2+ deposition, while simultaneously inhibits the removal of ferro senescent cells, leading to iron deposition in the CNS [75]. These iron-damaged genomes enable the mobilization of transposable elements (TEs), DNA segments that can extricate themselves from the genome and reinsert in the double helix at a different location [76]. TE mobilization has been associated with “fusogen storms”—the excessive release of fusion molecules from human endogenous retroviruses (HERVs), a large source of ancestral fusogens [77].

Despite being inserted in the human DNA, HERVs are mostly epigenetically suppressed by p53. Nonetheless, under pathological circumstances, these viral remnants can be expressed, being related to neurodegenerative disorders [78]. The virtual activation of HERVs have been implicated on the pathophysiology of COVID-19 [79]. SARS-CoV-2 can release fusogens by blocking p53, disinhibiting the transcription of syncytin-1, a member of the HERVs-W family that can activate several pro-inflammatory and autoimmune cascades in the brain, triggering neuropsychiatric pathology [80]. In HIV infection, the virus stimulates syncytin-1 transcription through interaction with TLR4 in primary astrocytes. This mechanism leads to activation and accumulation of Env proteins, which possess fusogenicity properties, as well as the capacity to activate the neuroimmune system, to damage oligodendrocytes and interfere with myelin regeneration [81, 82].

Interestingly, a previous study of our group showed that Spike-induced cognitive impairment in mice triggers innate immunity activation through TLR4, culminating in microgliosis, neuroinflammation, and synaptic pruning [33]. Remarkably, we also found plasma NfL increase in mice with Spike-induced cognitive impairment and this mechanism was dependent on TLR4 activation, because early TLR4 inhibition mitigated changes in NfL levels. At the time, we validated our preclinical findings by identifying an increased expression of TLR4 and high risk for cognitive impairment after SARS-CoV-2 infection on a cohort of patients with mild COVID-19 carrying the GG genotype of the TLR4-2604G > A (rs10759931) variant, when compared with the GA genotype. The current study used the same cohort and showed similar results regarding plasma NfL levels increase in patients with long COVID cognition and fatigue symptoms. Taken together, our findings suggest that the complex crosstalk between intracellular iron and Ca2+ deposition, HERVs and TLR4 can lead to cell senescence and the development of neurological symptoms in patients with long COVID.

Other coronaviruses are known for causing demyelination, neurodegeneration, and cellular senescence, all of which accelerate brain aging and potentially exacerbate underlying neurodegenerative pathology [83]. The neuroinvasive potential of SARS-CoV-2 may result in senescence of several different CNS cell types, such as oligodendrocytes and astrocytes, which may compromise remyelination of axons and the BBB integrity, just as limit the distribution of metabolic substrates of neuronal networks [84]. Besides, the senescence of neural stem cells may prevent neurogenesis in hippocampus, critical to memory consolidation [72]. The pNfL might be able to express this process, as it has been associated with other demyelinating and neurodegenerative diseases [16, 17, 85], as well as cell senescence [86].

NfL has been found to be significantly increased in acute COVID-19 patients when compared to HC, regardless of the severeness of the disease, and the presence of major neurological symptoms, such as encephalopathy [24, 87, 88]. However, some studies observed the normalization of these NfL levels in the long-term long COVID evaluation [89]. The mechanism regarding acute COVID-19 pNfL levels evolves systemic hyperinflammation, hypoxia and BBB disruption, and may differ from post COVID-19 mechanism. Despite the difference, short-term longitudinal pNfL levels of post COVID-19 were nominally, but not significantly, higher in COVID-19 patients than corresponding baseline ones in HC [87, 90]. The present study aims to investigate the relationship between the plasma levels of neurofilament light chain in patients with post-acute neurological symptoms (fatigue, cognitive dysfunction, and anxiety) and matched control who presented mild acute COVID-19 and provide information about the potential of NfL as a prognostic biomarker in those cases.

In time, results in studies about the correlation between NfL and long COVID are controversial. One study with hundred patients with mild, moderate, and severe COVID-19 showed that, after six months, NfL concentrations had normalized, with no persisting group differences, and they found no correlation between persistent neurological symptoms and CNS injury biomarkers in the acute phase [89]. Nonetheless, the mentioned study has some limitations, such as: they didn’t try to correlate NfL levels in the follow-up with neurological symptoms, only with acute levels; their form of classification of long COVID was based solely in self-reported symptoms questionnaires, without a more objective approach; and HC were from after SARS-CoV-2 pandemic, making it hard to exclude the possibility of the HC being infected. Also, they didn’t individualize the long COVID neurological symptoms, that demonstrated an important difference in our study.

In this study, it was exhibited that pNfL levels are significantly higher in long COVID patients with mild acute COVID-19 with neurocognitive symptoms when compared to HC (*p* = 0.0031), what could indicate the ongoing CNS damage caused by SARS-CoV-2, directly or indirectly, even in patients acutely with mild disease.

Nonetheless, it was demonstrated a divergence among the neurocognitive symptoms and their respective influence on the CNS injury. Levels of pNfL were significantly higher in long COVID patients with cognitive impairment and fatigue when compared to long COVID patients without these symptoms, individually and combined (*p* = 0.0263; *p* = 0.0480; and *p* = 0.0031, respectively). The combined analysis of the cognitive impairment and fatigue symptoms with significant higher levels of pNfL indicated the synergism between symptoms in the influence of pNfL levels, increasing the statistical power of the results.

Fatigue and cognitive complains are considered the most common and debilitating symptoms of long-COVID, directly affecting the quality of life of these patients [91]. The FSS has been widely used in post COVID-19 fatigue assessment [40–43, 92]. Besides, primary findings in neurocognitive profile of post COVID-19 patients exhibit deficits in attention and processing speed, and aspects of executive function [43]. The SDMT is a neuropsychiatric test used to evaluate the above-mentioned cognitive aspects, thus, resulting in a more reliable assessment. In this study, 69.8% of patients presented chronic fatigue after SARS-CoV-2 infection according to FSS, while 65.1% of patients presented cognitive impairment in SDMT. Indeed, when compared cognitive test results in adults recovering from COVID-19 with non-COVID-19 cases, it was found to be significantly reduced the cognitive performance in the COVID-19 group [93].

Both fatigue and cognitive impairment has been shown to be prevalent, as well as to persist and potentially worsen over time, in contrast to other persistent symptoms which may be self-limiting, such as anosmia [94]. In this sense, these symptoms could reflect ongoing neuro-damage, demonstrated by pNfL.

Not only the presence of cognitive impairment and fatigue in long COVID associated with higher pNfL levels, but also the levels of cognitive lost and exacerbation of fatigue in the neurocognitive evaluation had a significative correlation with pNfL levels. SDMT score T results correlated negatively with higher pNfL levels (*p* = 0.0219), and, comparably, FSS results correlated positively with higher pNfL levels (*p* = 0.0255). Therefore, a poorer cognitive performance and worse fatigue status indicates greater CNS injury. Furthermore, this authenticates both SDMT and FSS as powerful tools for the investigation of the degree of ongoing neuro-damage in long COVID patients.

In a follow-up study prior to the COVID-19 pandemic, elevated levels of NfL in cognitively healthy adults showed an association with the development of mild cognitive impairment [94, 95]. Moreover, protein markers of neuronal dysfunction including NfL were shown to be significantly increased in neuronal-enriched extracellular vesicle of participants recovering from COVID-19 compared to historic controls, suggesting ongoing peripheral and neuroinflammation after COVID-19 infection that may influence neurological sequelae [96].

Anxiety and depression symptoms in long COVID patients were also evaluated in this study. A meta-analysis with 4318 COVID-19 patients presented a prevalence of depression and anxiety symptoms in 38% of the sample [47]. HADS is the most used self-reported scale in COVID-19 research for evaluating anxiety and depressive symptoms [45, 47, 48]. In this sample, anxiety had a prevalence of 52.4%, while depression was presented in 39.7% of the individuals. Nonetheless, despite the high frequency, this study provided an absence of association and correlation between the presence of anxiety and depression symptoms in long COVID patients with higher pNfL levels.

This study has some limitations, such as: the absence of neurocognitive evaluation and pNfL levels from before the SARS-CoV-2 infection; Limited sample, although the results were already promising despite the number of patients; Majority of female sample, since it is a voluntary sample, composed by patients of the long COVID ambulatory of both university hospitals.

## Conclusion

In this study, it was exhibited that pNfL levels are significantly higher in long COVID patients with mild acute COVID-19 and neurocognitive symptoms when compared to HC, besides the dissimilarity among the neurocognitive symptoms and their respective influence on pNfL levels. Levels of pNfL were significantly higher in long COVID patients with cognitive impairment and fatigue when compared to long COVID patients without these symptoms, individually and combined. Additionally, poorer cognitive performance and worse fatigue status correlated with higher pNfL levels. These results demonstrate the potential of pNfL as a biomarker of the CNS ongoing injury in long COVID patients with cognitive impairment and fatigue. This can contribute to a better understanding of the mechanism of long COVID and to identify who will need continuous monitoring and treatment support. Further studies are needed to elucidate the extension of the CNS damage and possible neurodegenerating consequences.

## Data Availability

The datasets generated and/or analyzed during the current study are available from the corresponding author on reasonable request.
